# Patient-derived ovarian cancer explants: preserved viability and histopathological features in long-term agitation-based cultures

**DOI:** 10.1038/s41598-020-76291-z

**Published:** 2020-11-10

**Authors:** Sofia Abreu, Fernanda Silva, Rita Mendes, Teresa F. Mendes, Marta Teixeira, Vítor E. Santo, Erwin R. Boghaert, Ana Félix, Catarina Brito

**Affiliations:** 1 iBET, Instituto de Biologia Experimental e Tecnológica, Apartado 12, 2780-901 Oeiras, Portugal; 2 Instituto de Tecnologia Química e Biológica António Xavier, Universidade Nova de Lisboa, Avenida da República, 2780-157 Oeiras, Portugal; 3 Centro de Estudos de Doenças Crónicas da Faculdade de Ciências Médicas, CEDOC-FCM-NOVA, Universidade Nova de Lisboa, R. Câmara Pestana 6, 1150-078 Lisbon, Portugal; 4 AbbVie, 1 North Waukegan Road, North Chicago, IL 60064-6098 USA; 5 IPOLFG, Instituto Português de Oncologia de Lisboa Francisco Gentil, R. Prof. Lima Basto, 1099-023 Lisbon, Portugal

**Keywords:** Cancer microenvironment, Cancer models, Cancer therapeutic resistance, Chemotherapy, Ovarian cancer

## Abstract

Ovarian carcinoma (OvC) remains a major therapeutic challenge due to its propensity to develop resistance after an initial response to chemotherapy. Interactions of tumour cells with the surrounding microenvironment play a role in tumour survival, invasion capacity and drug resistance. Cancer models that retain tissue architecture and tumour microenvironment components are therefore essential to understand drug response and resistance mechanisms. Herein, our goal was to develop a long-term OvC patient-derived explant (OvC-PDE) culture strategy in which architecture and cell type heterogeneity of the original tumour would be retained. Samples from 25 patients with distinct OvC types and one with a benign tumour, were cultured for 30 days in agitation-based culture systems with 100% success rate. OvC-PDE cultures retained the original tumour architecture and main cellular components: epithelial cells, fibroblasts and immune cells. Epithelial cells kept their original levels of proliferation and apoptosis. Moreover, the major extracellular components, such as collagen-I and -IV, were retained in explants. OvC-PDE cultures were exposed to standard-of-care chemotherapeutics agents for 2 weeks, attesting the ability of the platform for drug assays employing cyclic drug exposure regimens. We established an OvC-PDE dynamic culture in which tumour architecture and cell type heterogeneity were preserved for the different OvC types*,* replicating features of the original tumour and compatible with long-term drug exposure for drug efficacy and resistance studies.

## Introduction

Ovarian carcinoma (OvC) is the eighth most commonly occurring cancer in women. There were nearly 300,000 new cases in 2018^1^. Since OvC does not show specific symptoms, 59% of the diagnoses are made at an advanced stage, contributing to a high mortality rate^2^. Standard OvC treatment includes surgery and chemotherapy with platinum-based drugs (i.e. carboplatin) and taxanes (i.e. paclitaxel)^3^. In approximately 60% of cases, a complete response only relates to a median progression-free survival of 18 months. Re-treating these patients with the same drugs typically results in response rates of around 50%^4^. Intrinsic and acquired resistance to platinum-based therapy are the main reasons for the unsuccessful treatment of patients with OvC^5,6^.

Several reports were recently published uncovering the role of stroma in chemoresistance^7^. Chen et al. demonstrated that stroma-rich epithelial ovarian tumours are correlated with higher lymph node status, faster cancer progression, and recurrence. Furthermore, patients with stroma-rich tumours had reduced progression-free survival and overall survival^8^. Several authors reported that cancer associated fibroblasts (CAFs) produce glutathione which conjugates with active drugs thus diminishing their accumulation in cancer cells and contributing to chemoresistance^9,10^. On the other hand, this resistance mechanism to platinum-based chemotherapy can be abolished by CD8+ T cells, improving OvC patient survival^10^. Given the role of stroma and infiltrating immune cells in cancer progression and drug resistance^11^, anti-cancer drugs that target the tumour microenvironment are a current trend in cancer drug discovery and development^12^. Therefore, cancer cell models that represent the tumour microenvironment and that can recapitulate drug response and the molecular processes of drug resistance, become crucial^13–16^.

Ex vivo models, mostly represented by tissue slices^14,17^ and explants^15,18^, present clear advantages as they potentially maintain the spatial conformation of the tissue, heterogeneity and tumour stage^19^. However, major disadvantages of ex vivo models are the lack of reproducibility due to the natural heterogeneity of donor tissues, the lack of standardised readouts^20^ and the short-term maintenance of different cell phenotypes^19^. Cell viability has been reported to be limited to 4–7 days^21–23^. A potential cause for low viability is a limitation in the diffusion of nutrients and oxygen, since intact vascularity is absent^19,24^. The short duration of these cultures hinders the study of tumour progression and early stages of metastasis in ex vivo models, as well as evaluation of cyclic drug treatments to address underlying or acquired resistance mechanisms^25^. Patient-derived explants of OvC are scarcely described in the literature. Although the reason for this is unclear, it may be related either with technical difficulties specific for OvC samples culture or with the availability of OvC patient-derived material, which highly depends on hospital collaborations and patients’ consent^26,27^.

We hypothesised that dynamic culture systems would improve cell viability and phenotype ex vivo by favouring efficient diffusion of oxygen and soluble compounds. OvC patient-derived explants (OvC-PDE) from different subtypes were cultured under orbital shaking for a month, maintaining cell type heterogeneity and viability. As a proof-of-concept, OvC-PDE were challenged with standard-of-care chemotherapy drugs (carboplatin or paclitaxel) in a cyclic exposure regimen.

## Results

OvC samples were obtained after surgical resection of ovarian tumours with distinct pathologies. Table 1 lists the pathological types and grades of the 25 OvC employed in this study: 12 high- (HGSC) and 1 low-grade serous carcinomas (LGSC), 1 mucinous, 3 mucinous borderline, 4 endometrioid, 2 clear cell carcinomas, 1 undifferentiated tumour, 1 carcinosarcoma (in the sample collected for culture, the epithelial component presented HGSC architecture); a fibroma (benign) was also utilised (Table 1; Supplementary Table S1). The majority of the OvC (48%) were HGSC, consistent with the high frequency of this OvC type^28^.

**Table 1:** Clinical pathological annotation of ovarian tumour samples.

Age		
Medium age (years)	68	
Age range	39–81	

	Number of cases (N = 26)	Percentage (%)
< 60	7	27
≥ 60	19	73
Histopathology diagnosis		
High Grade Serous carcinoma	12^a^	46
Endometrioid carcinoma	4	15
Mucinous borderline tumour	3	11
Clear cell carcinoma	2	8
Undifferentiated carcinoma	1	4
Carcinosarcoma	1	4
Mucinous carcinoma	1	4
Low Grade Serous carcinoma	1	4
Fibroma (benign tumour)	1	4

FIGO staging	Number of cases (N = 25)	Percentage (%)
Stage I	8	32
Stage II	1	4
Stage III	16	64

^a^4 cases with chemotherapy treatment before surgery.

**Figure 1:**
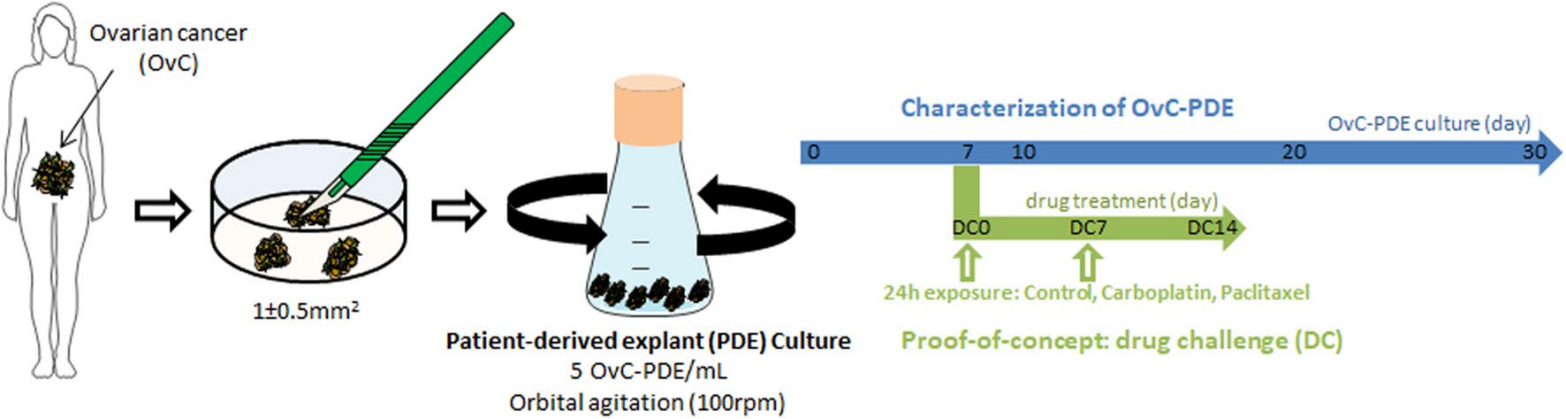
Schematic representation of the strategy pursued to establish long-term OvC-PDE cultures. This scheme was created using elements from Servier Medical Art (https://www.smart.servier.com), licensed under CC BY 3.0 (https://creativecommons.org/licenses/by/3.0/).

All samples were processed for OvC-PDE culture, as described in detail in the methods section (Fig. 1). Immediately after dicing the tumour, explants were incubated with fluorescein diacetate (FDA) and propidium iodide (PI) to assess viable and dead cells, respectively. Typically, dead cells were observed in the outer cell layer, probably a consequence of the mechanical processing. However, over culture, these cells dissociated from the explant, thus obtaining highly viable explants throughout one month of culture (Supplementary Fig. S1A, Fig. S2A). OvC-PDE cultures derived from serous carcinoma and carcinosarcoma (of which the epithelial component was exclusively composed of HGSC) had an average explant area of 1.0 ± 0.5 mm^2^ (OVC12, OVC14, OVC15, OVC16, OVC17, OVC20 and OVC23, Supplementary Fig. S1B), whereas OvC-PDE cultures derived from mucinous tumours and clear cell carcinomas were composed of larger explants, with a more heterogeneous size distribution (2.9 ± 1.0 mm^2^, OVC11, OVC13, OVC21 and OVC25, Supplementary Fig. S1B). After 10 days in culture, we observed a significant decrease in explant size (0.7 ± 0.3-fold relative to the initial size at day 0), probably corresponding to the dead outer layer that dissociated from each explant. By day 30 of culture, average explant size was 0.3 ± 0.1 times the initial size (p < 0.0001 between day 0 and day 30 of culture, Supplementary Fig. S2B) and with higher homogeneity as indicated by the different variance of the samples (F test, p < 0.05). Additionally, explant concentration increased overtime, reaching a 2.8 ± 1.5-fold increase relative to the initial concentration (Supplementary Fig. S2C).

**Figure 2:**
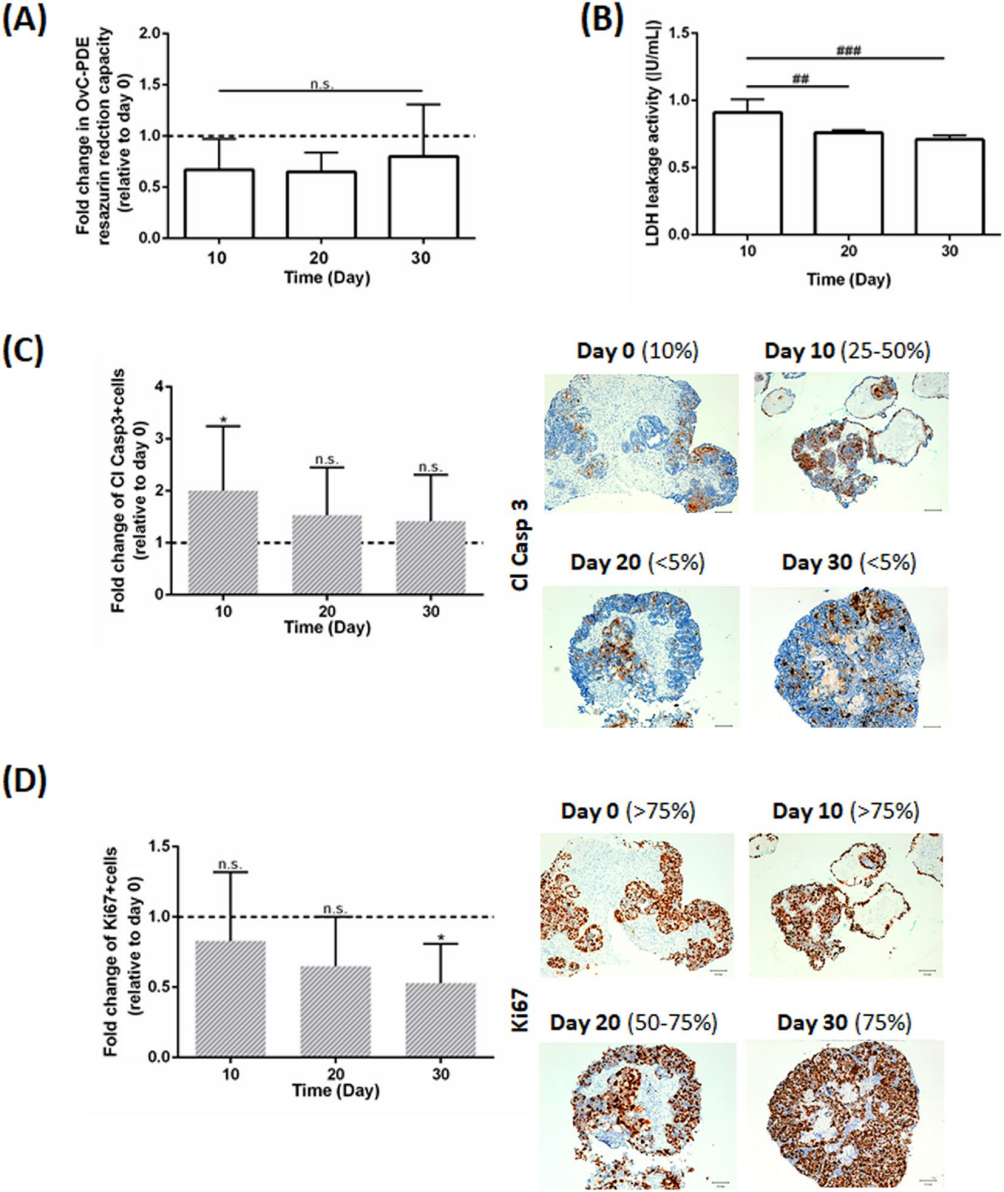
OvC-PDE cultures were derived from different subtypes and remained viable for 30 days. (**A**) Resazurin reduction capacity of the OvC-PDE along culture time relative to day 0. Data is presented as mean ± SD (N ≥ 7). Two-way ANOVA statistical test (Tukey’s multiple comparison test) was applied to compare the mean values of resazurin reduction capacity along culture period relative to day 0 (*) and between timepoints (#). Statistical analysis was carried out using GraphPad Prism 6 Software; **,^##^(p < 0.01) and ^###^(p < 0.005); (**B**) LDH activity in the OvC-PDE culture supernatants. Data is presented as mean ± SD (N = 5). One-way ANOVA statistical test was applied to compare LHD activity along culture period. Statistical analysis was carried out using GraphPad Prism 6 Software. ^##^(p < 0.01); ^###^(p < 0.001); (**C**) quantification of apoptosis (cleaved caspase 3^+^ cells) by immunohistochemistry (N = 15) and representative images (OVC15) of OvC-PDE cultures collected at days 0, 10, 20, and 30. Scale bars represent 100 µm. One-way ANOVA statistical test was applied to compare cleaved caspase 3^+^ cells along culture time versus day 0. Statistical analysis was carried out using GraphPad Prism 6 Software. *(p < 0.05); *n.s*. not significant; (**D**) quantification of proliferation (Ki67^+^ cells) by immunohistochemistry analysis (N = 13) and representative imagens (OVC15) of OvC-PDE maintained in agitation-based cultures and collected at days 0, 10, 20, and 30. Scale bars represent 100 µm. One-way ANOVA statistical test was applied to compare Ki67^+^ cells along culture time versus day 0. Statistical analysis was carried out using GraphPad Prism 6 Software. *(p < 0.05); *n.s*. not significant.

All OvC types and the fibroma maintained high cell viability during the culture period. By live/death analysis, we observed homogenous green fluorescence in OvC-PDE cultures, indicating high cell viability (Supplementary Fig. S2A). After 30 days in culture, OvC-PDE cultures remained metabolically active, as assessed by resazurin reduction capacity (0.8 ± 0.5-fold change relative to day 0 of culture, Fig. 2A), and maintained the original apoptosis levels (1.42 ± 0.89-fold change in cleaved caspase-3+ cells relative to day 0 of culture, Fig. 2C). Importantly, after 20 days in culture, no statistical significant difference was observed in terms of proliferation (0.65 ± 0.35-fold change in Ki67^+^ cells relative to day 0 of culture, Fig. 2D). After 30 days, OvC-PDE cultures maintained a high cell proliferation rate, despite the slight decrease observed (0.53 ± 0.28-fold change in Ki67^+^ cells relative to day 0 of culture, Fig. 2D). Curiously, there was a transient increase in cell death (necrosis and apoptosis) up to day 10 of culture, detected by a peak in LDH activity in the culture supernatant (Fig. 2B) and a twofold increase of cleaved caspase-3 (Fig. 2C), respectively and then the levels returned to values similar to day 0 of culture up to 30 days of culture.

**Figure 3:**
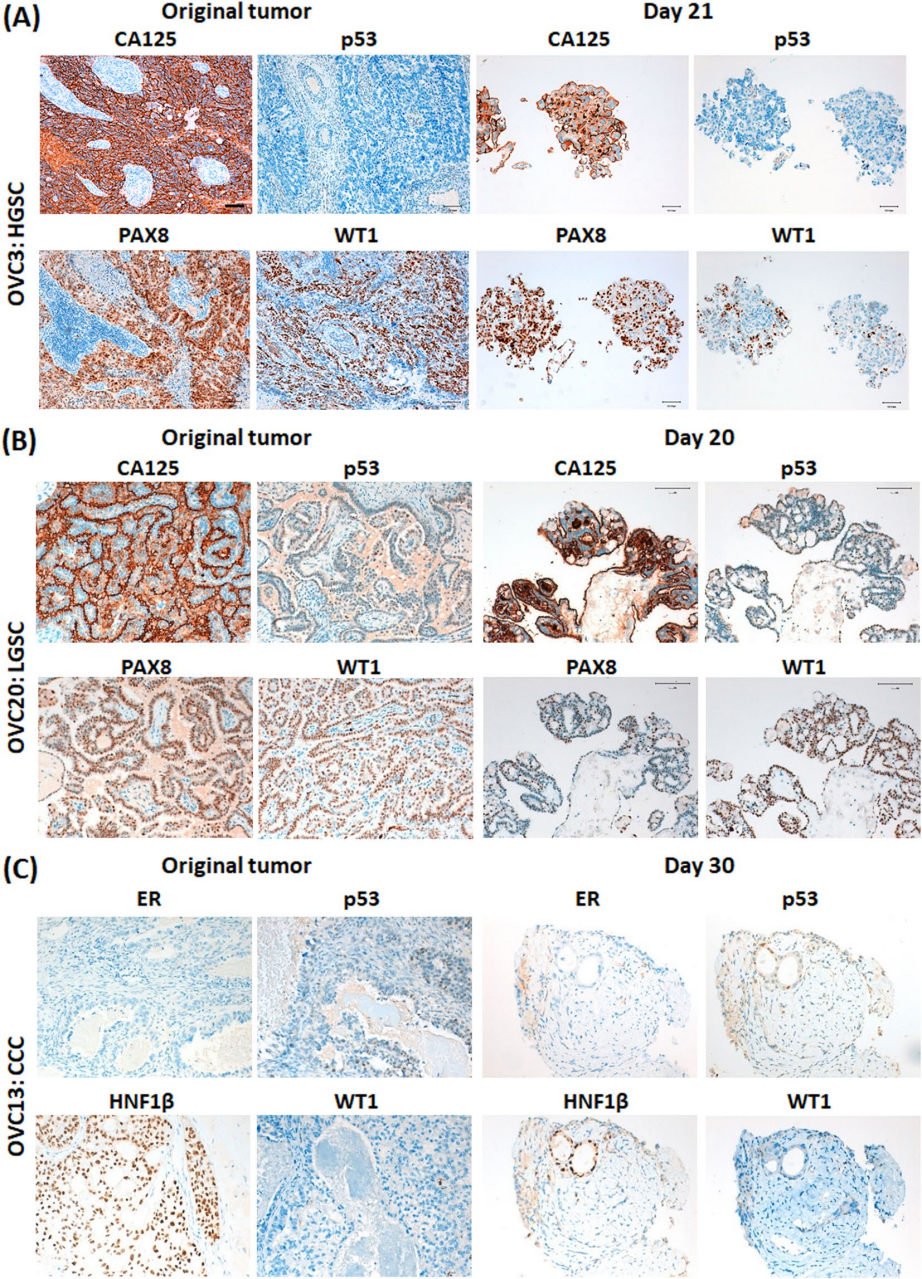
OvC-PDE cultures maintained a phenotype similar to the original tumour for 30 days. Immunohistochemistry of cross-sections of tumour samples and OvC-PDE cultures (representative images, N = 3) of (**A**) High Grade Serous Carcinoma (HGSC), OVC3, (**B**) Low Grade Serous Carcinoma (LGSC)**,** OVC20 and (**C**) Clear Cell Carcinoma (CCC), OVC13 at day 21, 20 and 30, respectively. Scale bars 100 µm. *CA125* Cancer Antigen 125, *p53* tumour suppressor, *PAX8* Paired-box gene 8, *WT1* Wilms tumour protein 1, *ER* Oestrogen Receptor, *HNF1β* Hepatocyte Nuclear Factor 1β.

Moreover, we compared the antigen profile of OvC-PDE cultures with the original tumour. Detection of the OvC marker Cancer Antigen 125 (CA125)^29^ was maintained in HGSC- and LGSC-derived OvC-PDE cultures (Fig. 3A,B, respectively). PAX8 and WT1, HGSC markers^30^, were also detected (Fig. 3A,B). For OVC3, p53 was not detected in both the original tumour and the OvC-PDE, suggesting that this particular tumour was a p53 null expression (nonsense mutation of TP53, Fig. 3A). In clear cell carcinoma, the patterns of detection of oestrogen receptor (ER), HNF1β and WT1 were also similar to the original tumour, despite variations in intensity (Fig. 3C).

**Figure 4:**
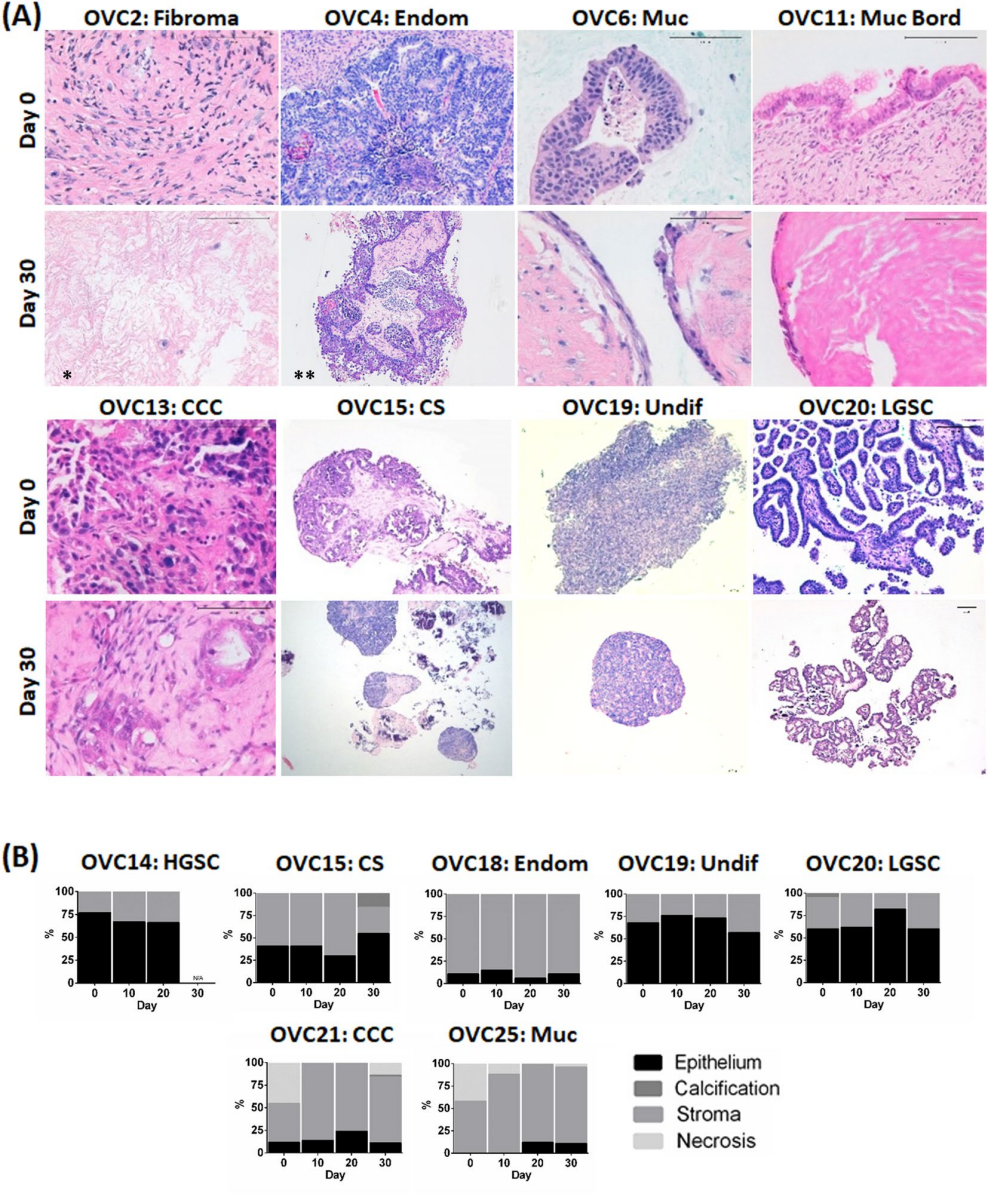
OvC-PDE cultures maintained epithelial and stromal cell populations similar to the original tumour. (**A**) Haematoxylin and eosin (H&E) staining of cross-sections of OvC-PDE cultures derived from OvC of diverse subtypes (representative images, N = 25) and a fibroma, along culture time. Scale bars represent 100 µm; *day 100; **day 21; (**B**) percentage of epithelium (black), stroma (grey), calcification (dark grey), necrosis (light grey) along OvC-PDE culture time in different OvC subtypes (N = 7); *CS* carcinosarcoma, *CCC* clear cell carcinoma, *Endom* endometrioid carcinoma, *HGSC* high grade serous carcinoma, *LGSC* low grade serous carcinoma, *Muc* mucinous carcinoma, *Muc bord* mucinous borderline tumour, *Undif* undifferentiated carcinoma.

**Figure 5:**
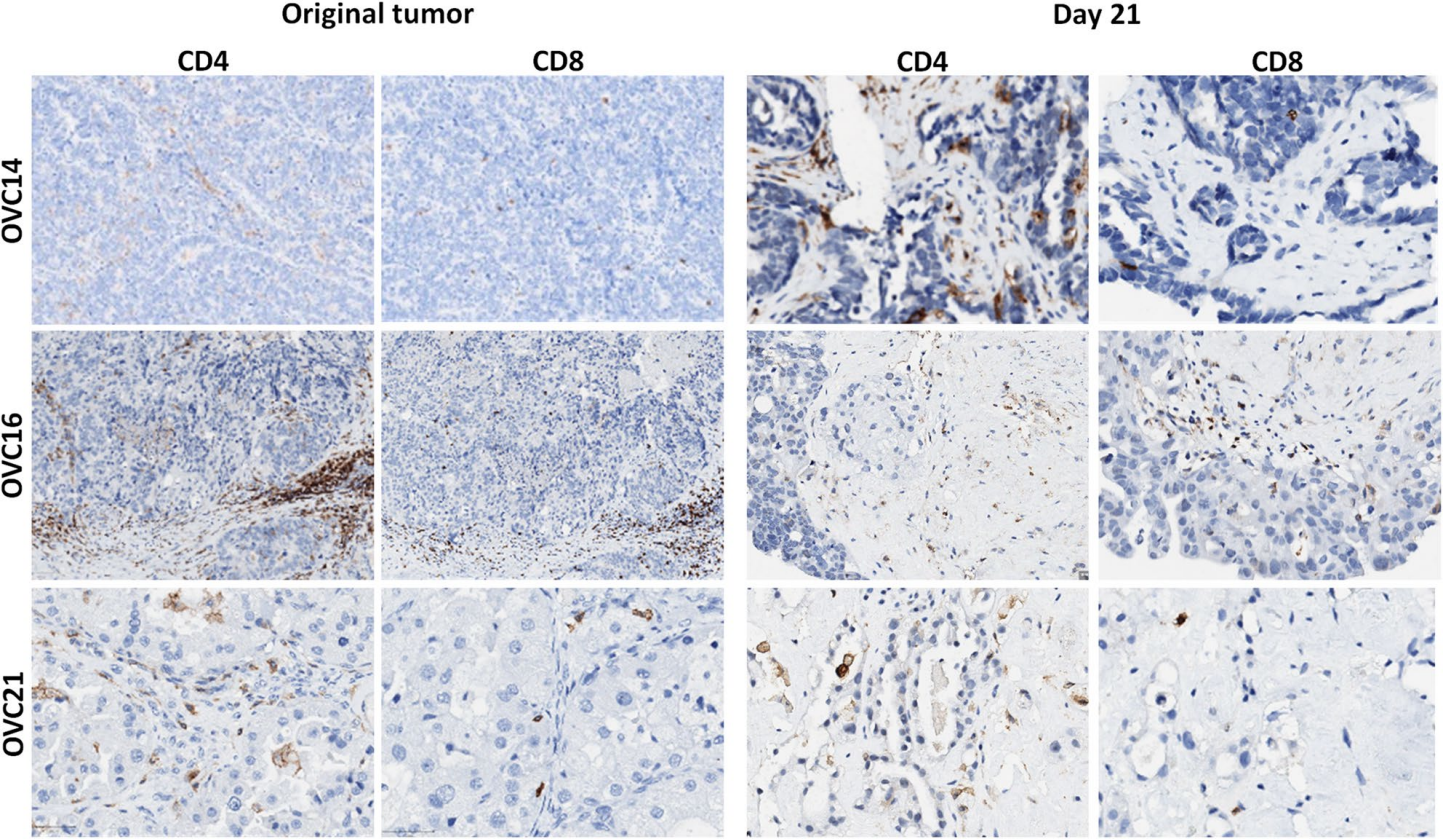
OvC-PDE cultures retained the T cell populations of the original tumour. Immunohistochemistry of cross-sections of tumour samples and OvC-PDE cultures at day 21 from OVC14, OVC16 and OVC21 (representative images, N = 3). Scale bars represent 100 µm.

Epithelial cells and fibroblasts were identified after one month of OvC-PDE culture of all the tumours employed in the study, by morphologic inspection of H&E staining (Fig. 4A). Importantly, the ratio of the two cellular compartments was also maintained in all samples analysed (Fig. 4B). Furthermore, OvC-PDE derived from tumours with immune infiltrate preserved the tumour infiltrating lymphocytes (TILs), namely CD4+ and CD8+  T cells (Fig. 5). The immune infiltrate of the OVC16 tumour contained B cells and macrophages; these were also detected in OvC-PDE culture, by IHC for CD20 and CD68, respectively (Supplementary Fig. S3A). Moreover, in one of the OvC-PDE cultures we evaluated collagen I and IV, two of the major extracellular matrix (ECM) components in OvC, as well as integrins β1 and β4, the cell surface receptors for collagen-I and laminin adhesion^31,32^. Labelling was similar in the original tumour and in OvC-PDE cultured for 30 days (Supplementary Fig. S3B). Sequential series of cross-sections of several cases were analysed by H&E; the phenotype and morphology within each individual OvC-PDE was homogeneous across its depth, suggesting that OvC-PDE did not present necrotic cores nor induced regionalization of specific cell types (Supplementary Fig. S4).

Overall, the data showed that OvC-PDE of approximately 1 mm^2^ can be maintained in agitation-based culture systems for at least 30 days, retaining features of the original tumour, such as architecture, cellularity (epithelial, stromal and immune infiltrate), as well as subtype-specific antigen profile, proliferation and apoptotic indexes.

**Figure 6:**
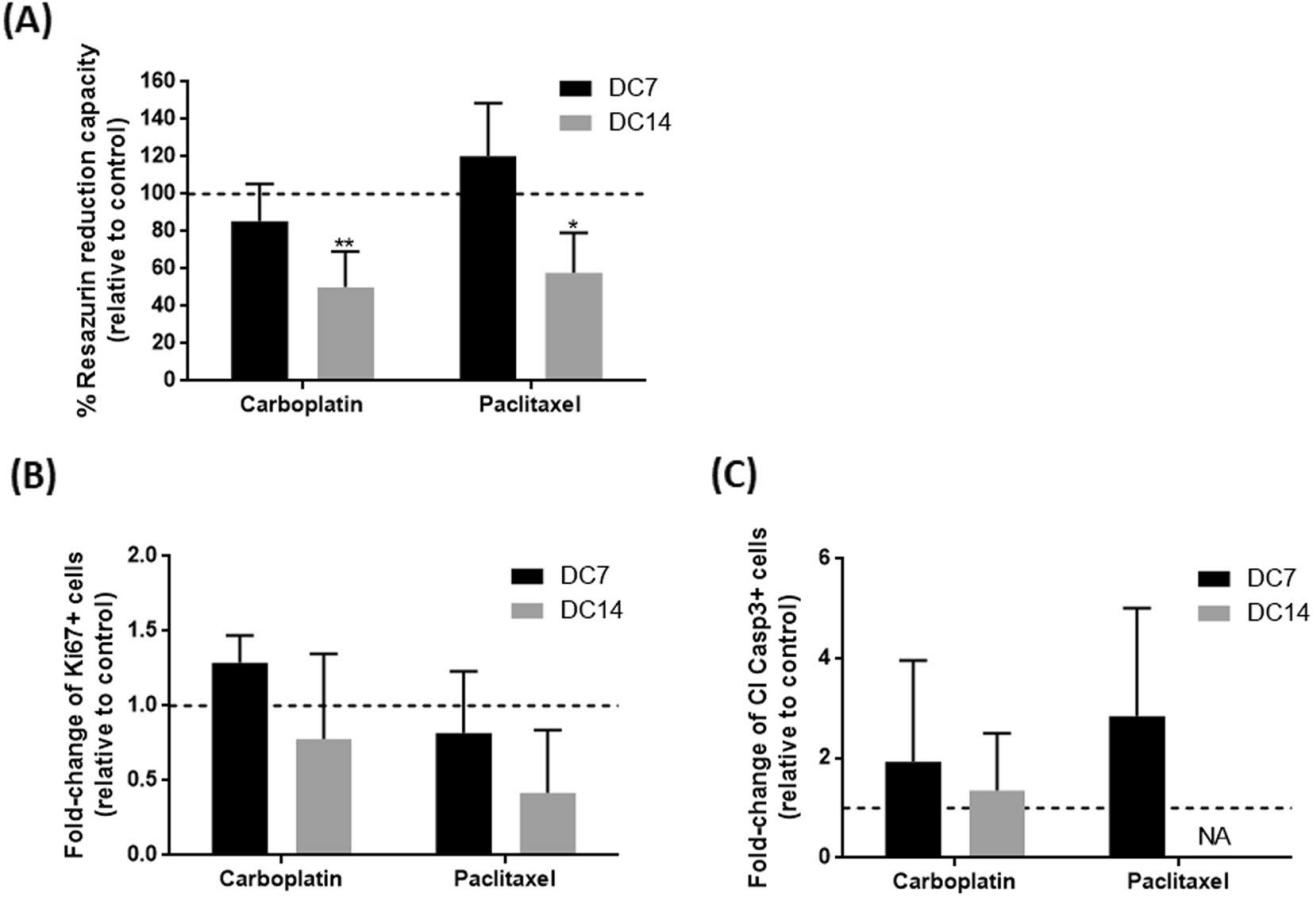
OvC-PDE cultures are amenable for ex vivo evaluation of chemotherapy efficacy. (**A**) Resazurin reduction capacity (% relative to control) of OvC-PDE cultures at day 0, 7 and 14 of drug challenge (DC, corresponding to day 7, 14 and 21 of culture) with 25 µg/mL carboplatin and 10 µg/mL paclitaxel. Two-way ANOVA statistical test (Tukey’s multiple comparison test) was applied to compare the mean values of resazurin reduction of OvC-PDE culture after each drug challenge versus control culture. Statistical analysis was carried out using GraphPad Prism 6 Software. *(p < 0.05) and **(p < 0.01); (**B**) proliferation (Ki67^+^ cells) and (**C**) apoptosis (cleaved caspase 3+ cells) quantification in cross-sections of OvC-PDE cultures challenged with chemotherapy agents. Data in (**A**–**C**) are presented as a mean ± SD (N = 4, OVC15, OVC16, OVC23 and OVC24).

As a proof-of-concept of the applicability of our OvC-PDE culture system to test repeated drug exposure, we challenged OvC-PDE cultures with standard-of-care chemotherapeutic agents (Figs. 1, 5). Resazurin reduction capacity of OvC-PDE challenged with carboplatin or paclitaxel, at the reported physiological peak plasma concentrations^33,34^, remained similar to the control after the 1st cycle of treatment (Fig. 6A). Only after the 2nd cycle, a significant reduction was observed relative to untreated control cultures, with a decrease in viability of 50 ± 19% (p < 0.01) for carboplatin and 58 ± 21% (p < 0.05) for paclitaxel (Fig. 6A). Drug-induced cell death was confirmed by immunohistochemistry analysis: we observed a decrease in epithelial cell content after 2 cycles of treatment, with lower levels of proliferative Ki67^+^ cells (0.78-fold change with carboplatin and 0.42-fold change with paclitaxel, relative to control, Fig. 6B, Supplementary Fig. S5A), although the levels of apoptotic cleaved caspase-3+ cells were similar to the control condition (1.15-fold change with paclitaxel, Fig. 6C, Supplementary Fig. S5A). Moreover, 2 additional OvC-PDE were exposed to the same drug exposure regimen, at the same drug concentrations and 10 times higher ones. At the higher drug concentrations, reazurin reduction capacity dropped to residual levels (Supplementary Fig. S5B), suggesting a dose–response behaviour of the drugs in the OvC-PDE setup.

## Discussion

OvC remains a major therapeutic challenge due to its propensity to develop resistance after an initial response to chemotherapy^35^. Interactions of tumour cell with the surrounding microenvironment play a role in tumour survival, invasion capacity and drug resistance^36,37^. Cancer cell models that retain tissue architecture and tumour microenvironment components are therefore essential to understand drug response and resistance mechanisms.

Herein, we describe a novel culture strategy that improves the longevity and preserves the histopathological features of OvC explants, by taking advantage of agitation-based culture systems^38^. Our platform sustained high cell viability levels and maintained the original tumour phenotype for at least 30 days with 100% success rate. Importantly, this strategy was successful for the culture of eight subtypes of OvC, from type I, type II and borderline tumours^39,40^. Interestingly, the methodology also worked for a non-malignant tumour (fibroma), with the preservation of stromal cells.

OvC-PDE cultivated up to one month retained metabolic activity, proliferation rates and apoptotic levels similar to the original tumour without formation of hypoxia gradient and necrotic cores. The 30 days of culture reported herein are an improvement over the commonly reported 2 to 7 days^18,20,21,41^. The transient increase in cell apoptosis observed during the first 10 days of culture suggests a period of adaptation to culture conditions after the sample's mechanical processing. We explored agitation-based systems, reported to improve the diffusion of oxygen and soluble factors (nutrients, metabolic waste products, soluble factors and cytokines)^38,42–44^. Dynamic culture systems have been previously proposed for culture of ex vivo cancer models^19,38,45,46^. These studies report improvements in cellular physiology and viability^47^ over static systems. For instance, Van der Kuip et al*.* described a strategy for the culture of breast cancer precision cut thin slices based on orbital shaking, in which cells remained viable and proliferative for at least 4 days^48^. Naipal et al*.* used a similar strategy and found that the continuous movement promoted nutrient exchange, leading to a higher percentage of proliferative cells and extended culture viability (7 days), in comparison to stationary conditions^21^. We generated OvC-PDE of an average size of 1 mm^2^. For mucinous and clear cell carcinoma, OvC-PDE were larger and had a more heterogeneous size distribution in comparison with their counterparts derived from serous carcinomas and a carcinosarcoma with serous component. This could be correlated with the presence of mucus that created thread-like structures. Naipal et al. reported difficulties in homogeneous processing of soft, mucinous and fibrous tissue^21^.

We hypothesised that thick OvC-PDE could have advantages over thin slices by retaining endogenously secreted soluble factors and sustaining retention of ECM, contributing to sustain cellular crosstalk within the tissue^49,50^. ECM components have been gaining attention due to their important role in tumour growth and progression^51^. Tumour architecture and stroma composition, but also their secreted factors, have been recognised as major players in the establishment and progression of cancer cells^49,50^. Specifically, in the OvC context, CAFs are known to express and secrete high levels of chemokine (CXC motif) ligand CXCL‐1, which binds to its receptor CXCR2, highly expressed on OvC cells, thus promoting cell proliferation^52^. CAFs also secrete high levels of Interleukin 6 (IL‐6) and cyclooxygenase 2 (COX‐2), factors correlated with inflammation, angiogenesis and proliferation support, this way circumventing apoptosis and promoting tumour progression^52,53^. In addition, OvC CAFs are the main source of ECM components, such as collagens type I, III and V and fibronectin and matrix metalloproteinases (MMPs)^54^.

In fact, our culture system allowed the retention of epithelial, stromal and immune compartments of the original tumours, by applying a common strategy and a basal medium composition to the different subtypes. Interestingly, when ECM components were analysed in one of the OvC-PDE, collagen-I and -IV, as well as the ECM receptors β1 and β4 integrin^55^ were strongly detected after one month of culture. To our knowledge, this is the first long-term ex vivo study reporting the maintenance of these OvC microenvironment components. Recently, Hill et al*.*^56^ and Kopper et al*.*^27^ reported short and long-term organoid platform for OvC, respectively. Organoids recapitulate subtype-specific histological and genomic features and allow for the expansion of tumour cells. However, these models are derived from malignant epithelial cells, lacking the tumour microenvironment^27,56^.

A long-term model of OvC that retains the tumour microenvironment and patient-specific features allows to explore unaddressed disease mechanisms, mainly related with OvC tumour progression and metastasis formation, as well as to perform resistance studies through the evaluation of cyclic drug treatments. To demonstrate the potential use of our model as a drug assay platform, we challenged OvC-PDE with two cycles of standard-of-care chemotherapeutic agents. Exposure to the reported physiological peak plasma concentrations of carboplatin and paclitaxel^33,34^ led to a significant reduction in cell viability only after the second cycle of treatment. A tenfold higher drug concentration led to total cell death, highlighting the potential influence of the drug exposure regimen in drug efficacy. We also assessed proliferation and apoptosis after the drug challenge by immunohistochemistry (IHC), as the latter is broadly used in the clinics as a readout of drug response. Although not significant, both drugs tended to reduce the epithelial compartment and the remaining epithelial cells decreased their proliferation. Additionally, we observed an increased apoptosis upon the second cycle. These results are aligned with previous reports, where carboplatin was used in the same concentration range, and with clinical response; in neoadjuvant therapy at least three cycles are preconized^57,58^.

In the long-term perspective, one can envision the utilisation of the OvC-PDE to assess patient chemosensitivity, to assist in therapeutic decisions. Nonetheless, this implies the previous evaluation of the degree of correlation between patient response/clinical outcome and the ex vivo response, which requires longer follow-up time, as well as increased cohort size. Some correlations studies using ex vivo platforms are already described. For example, a platform based machine-learning algorithms that combined the results in ex vivo models with clinical information (patient history, tumour stage and pathology from biopsies) to predict the clinical outcome after treatment^16^.

Our model can also be used to explore and evaluate the efficiency of new compounds in preclinical phase or assessing drug combinations, although it cannot accommodate high-throughput screening campaigns. The fact that proliferation and apoptosis rates within OvC-PDE are similar to the original tumour rates impairs propagation of the material. Currently, the gold standard in cancer drug discovery is the xenograft mouse model^20,59–61^. Although this model preserves tumour cell viability and architecture^62^, patient-derived xenografts (PDX) present a low engraftment rate^63,64^ and along passages the human tumour microenvironment is replaced by mouse components^65–67^. Moreover, OvC PDX have been reported to exhibit a significant loss of steroid hormone receptors and altered expression of immunoresponsive genes^68^. PDX are mostly generated in immunocompromised mice, which lack the contribution of the immune system^69^. For evaluation of immunotherapies, fresh PDX which retain patient’s immune cells^70^, and humanised mice have been developed^71^. Nevertheless, some limitations remain such as missing cross-reactivity of cytokines and growth factors between species, resulting in graft-versus-host disease (GVHD) typically within 4 weeks^72^. In addition, these models demand considerable costs and are extremely laborious^73^. OvC-PDE retained viable tumour infiltrating lymphocytes (TILs), presenting both CD4+ and CD8+ T cells. In accordance, in a study of 186 samples of advanced-stage OvC, it was found that around half of the patients had CD3+ TILs, presenting both CD4+ and CD8+ T cell populations^74^. In the future, it will be interesting to evaluate the functionality and immunosuppressive status of the TILs present within OvC-PDE cultures to evaluate their real potential for immunoncology studies.

## Conclusions

In this work, we provided experimental evidence of the feasibility to culture OvC-PDE in agitation-based culture systems for at least 1 month. The main OvC types were successfully cultured as OvC-PDE and we could establish and maintain these cultures up to 30 days with high cell viability and with proliferation and apoptosis levels similar to the original tumour. OvC-PDE cultures preserved the histopathological features of original tumours, with maintenance of the epithelial and stromal components, as well as the immune infiltrate. As a proof-of-concept of the applicability of the model for repeated-dose drug assays, OvC-PDE cultures were challenged weekly with standard-of-care chemotherapy. To sum up, this is the first report of one-month long ex vivo patient-derived OvC model with preservation of microenvironment features, compatible with cyclic drug challenge. With such characteristics, this model system can contribute to fundamental research of OvC but also for precision medicine approaches.

## Materials and methods

### Sample collection and processing of tumour tissue

Fresh human tumours were collected from patients with signed informed consent that underwent surgery at the Instituto Português de Oncologia de Lisboa, Francisco Gentil (IPOLFG). Samples were named chronologically from OVC1 to OVC27 (Supplementary Table S1); sample OVC22 was not included in the study since it was diagnosed as a breast cancer metastasis. Tumour specimens were transported in Dulbecco’s Modified Eagle’s Medium (DMEM, Cat. Number 41965-039, Gibco) from the surgery room to the laboratory. Tumour samples were processed up to 4 h after surgery. After washing with Dulbecco’s Phosphate Buffered Saline (DPBS, Gibco) and mass determination, tumour samples were immersed in Culture Medium, composed by DMEM supplemented with 10% Fetal Bovine Serum (FBS, 10270-106, Gibco) and 1% PenStrep (P/S, penicillin and streptomycin, Cat. Number 15140-122, Gibco). Samples were cut into fragments of approximately 1.0 mm^2^, using two disposable sterile scalpels (Cat. Number 0503, Swann-Morton).

### Establishment of patient-derived explant cultures (OvC-PDE)

OvC-PDE cultures were maintained at 5 explants/mL, in 20 mL culture medium (DMEM supplemented with 10% FBS and 1% P/S), in 125 mL regular Erlenmeyer shake flasks (Corning). Cultures were kept under orbital shaking (IKA KS 260 basic) at 100 rpm, to prevent adhesion and increase oxygen and nutrients diffusion, in an incubator (Nuaire US Autoflow) at 37 °C, 5% CO_2_ in air. Cultures were maintained up to 30 days. Culture medium was renewed once a week (50% of the total volume). OvC-PDE were collected at day 0 (surgery day, after sample processing), 10, 20 and 30 of culture. Due to restriction of primary material, and the destructive nature of several endpoints, not all OvC-PDE could be used for all read-outs, at all timepoints: all OvC-PDE cultures were evaluated by H&E in all timepoints; as for the remaining read-outs, they were selected taking into account the work phase and availability of material, as identified in each method.

### Live/dead assay

The cell viability was assessed during culture time using fluorescein diacetate, a cell permeant esterase substrate (fluorescein diacetate—FDA, 10 µg/mL in DPBS, Molecular Probes), to label live cells and propidium iodide (PI, 2 µg/mL in DPBS, Molecular Probes), a cell impermeant DNA dye to stain dead cells^75^. Herein, 3–5 explants were collected at day 0, 10, 20 and 30 of each OvC-PDE culture analysed (N = 15), incubated for 5 min at room temperature with a solution with FDA/PI and visualised using a fluorescence microscope (DMI6000 Leica Microsystems CmBH, Wetzlar, Germany). Image analysis was performed with open access Image J Software (Rasband, WS, ImageJ, U.S. National Institutes of Health, Bethesda, MD, USA, https://imagej.nih.gov/ij/, 1997–2012).

### Resazurin reduction capacity

Resazurin reduction capacity of cells present in the explants was assessed using the PrestoBlue Cell Viability Reagent (A13262, Invitrogen). The active ingredient of PrestoBlue reagent (resazurin) is a non-toxic and non-fluorescent dye, that when in contact with a viable cell is reduced, becoming red-fluorescent resorufin^76^. At days 0, 10, 20 and 30 of each OvC-PDE culture analysed (N = 7–8), 1 mL of culture suspension (on average, 5 explants) was collected in triplicate, and incubated with PrestoBlue reagent (diluted 1:10) during 1 h at 37 °C. After this, supernatants of triplicates were collected to a 96-well black fluorescence reading plate (Corning) and fluorescence reading was performed (Infinite 200 PRO NanoQuant plate reader, TECAN), setting the excitation wavelength at 570 nm and emission wavelength at 590 nm.

### LDH leakage

Lactate dehydrogenase (LDH) is a soluble cytoplasmic enzyme that is released in culture supernatant when the cell membrane is damaged, allowing the monitoring of cell death^77^. For samples preparation, 0.5 mL of culture supernatant of each OvC-PDE culture analysed (N = 5) were collected in triplicate, centrifuged (Eppendorf) at 1000*g* for 5 min. The supernatant was incubated with NADH (Sigma-Aldrich) in 96-well plate and absorbance reading was performed in TECAN plate reader (Infinite 200 PRO NanoQuant), at 340 nm and 37 °C, with kinetic cycle every 10 s, for 2 min. After addition of sodium pyruvate (Sigma-Aldrich), absorbance was measured again using the same parameters. Control data shown that the LDH is a stable enzyme in OvC-PDE culture supernatant at 37 °C for 7 days (data not shown).

### Explant surface area

Explant surface area was calculated at days 0, 10, 20 and 30 of culture. Briefly, OvC-PDE were collected (N = 8–12) and observed by phase-contrast microscopy (DMI6000 Leica Microsystems CmBH, Wetzlar, Germany). The dimensions were measured using open-access Image J Software by manually defining the fragment boundaries for automatic calculation of area (mm^2^). Data is presented as mean ± standard deviation from at least 15 different explants per condition.

### Explant concentration

Explant concentration (explant/mL) was calculated by sampling triplicates of culture at days 0, 10, 20 and 30 (N = 6–8). OvC-PDE number was counted under a phase contrast microscope. Data is presented as mean ± standard deviation from at least 15 different explants per condition.

### Morphology and immunohistochemistry (IHC) analysis

OvC-PDE samples (N = 24) were fixed in formaldehyde and embedded in paraffin. Sections (3 µm) were stained with hematoxylin and eosin staining (H&E, Hematoxylin, Cat. Number CS700, Dako; and Eosin, Cat. Number CS701, Dako). Quantification of epithelial and stromal compartments, calcification and necrosis was performed by a pathologist, data is presented in % of each area over the total OvC-PDE area, using open source Image J Software.

For IHC, all antibody dilutions were made in Antibody Diluent Reagent Solution (Cat. Number 003218, Life Technologies). IHC of sections was performed in a BenchMark ULTRA IHC/ISH Automatic staining platform (Ventana Medical Systems) using OptiView DAB IHC Detection Kit (Ventana Medical Systems) with diaminobenzidine as the chromogen to detect antigen expression. Image acquisition was performed in Digital Microimaging Device Leica DMD108 (version 1.15 Build 704, Leica Microsystems).

OvC markers were detected (N = 3) with anti-CA125 (OC125, Cat. Number 325 M-16, Cell Marque, dilution 1:150 for 12 min; pretreatment ULTRA CC1-24 min), anti-PAX8 antibody (clone MRQ-50, Cat. Number 363 M-16, Cell Marque, dilution 1:100 for 32 min; pretreatment ULTRA CC1-56 min), anti-WT1 antibody (clone 6F-H2, Cat. Number M3561, DAKO, dilution 1:100 for 32 min; pretreatment ULTRA CC1-56 min), anti-p53 antibody (clone DO7, Cat. Number 453 M-96, Cell Marque, dilution 1:150 for 16 min; pretreatment ULTRA CC1-36 min), anti-ER antibody (clone SP1, Cat. Number 790-4324, pre-diluted for 28 min; pretreatment ULTRA CC1-56 min) and anti-HNF1beta antibody (Cat. Number HPA 002083, Sigma, dilution 1:1000 for 28 min; pretreatment ULTRA CC1-56 min). The clinical criteria implemented at the Pathology Department of the Portuguese Institute of Oncology of Lisbon Francisco Gentil (IPOLFG) were followed, based on the assessment of malignant epithelial cells positive for the markers relative to the total number of malignant epithelial cells.

Immune cell subpopulations were detected by IHC; CD4 and CD8-positive T cell subsets were identified (N = 3) with anti-CD4 antibody (clone SP35, Cat. Number 790-4423, pre-diluted for 16 min; pre-treatment ULTRA CC1-32 min) and anti-CD8 antibody (clone SP57, Cat. Number 790-4460, pre-diluted for 16 min). In one OvC-PDE culture, markers for macrophages (CD68+, anti-human CD68, clone PG-M1, dilution 1:3000, Cat. Number M0876, Dako), T cells (CD3+, anti-human CD3, clone LN10, dilution 1:200, Cat. Number NCL-L-CD3-565, Novocastra, Leica Microsystems) and B cells (CD20+, anti-human CD20, clone L26, dilution 1:1000, Cat. Number M0755, Dako) were also employed.

Proliferation and apoptosis status were assessed (N = 13) by labelling Ki67 (anti-Ki67, clone 30-9, pre-diluted, Cat. Number 790-4286) and cleaved caspase-3 (cleaved caspase-3, clone Asp 175, dilution 1:100, Cat. Number 9661, Cell Signaling), respectively. Quantification of Ki67^+^ and cleaved caspase-3+ cells was performed by a pathologist; at least 100 cells were counted in the original tumour and all malignant cells were counted in OvC-PDE; data is presented in % of positive cells over the total OvC-PDE cell number. Classification not applicable (N/A) indicates that a score could not be given due to insufficient amount of histological material for quantitative analysis (explant number < 5).

Extracellular matrix was characterised (N = 1) by anti–collagen I antibody (clone EPR7785, Cat. Number ab 138492, Abcam, dilution 1:300 for 20 min; pretreatment ULTRA CC1-16 min); anti-collagen IV antibody (clone CIV22, Cat. Number M0785, DAKO, dilution 1:10 for 20 min; pretreatment ULTRA CC1-16 min); anti-integrin β1 antibody (clone D2E5, Cat. Number 9699S, Cell Signaling, dilution 1:100 for 28 min; pretreatment ULTRA CC1-92 min) and anti-integrin β4 antibody (Cat. Number 036348, Human Protein Atlas, dilution 1:200 for 28 min; pretreatment ULTRA CC1-56 min).

### Drug challenge of OvC-PDE with chemotherapeutic agents

Ex vivo chemotherapy challenge was performed in explants covering three OvC subtypes: 2 high-grade serous carcinomas (OVC16/23), 1 endometrioid tumour (OVC24), 1 carcinosarcoma (OVC15), N = 4. OvC-PDE at day 7 of culture (Drug Challenge Day 0, DC0) were exposed to 25 µg/mL carboplatin (Fresenius Kabi) or 10 µg/mL paclitaxel (Fresenius Kabi). The drug concentrations were determined based on previously reported peak plasma concentrations (14.7–28.3 µg/mL for carboplatin^33^ and 10 µg/mL for paclitaxel^34^). OvC-PDE were exposed to 2 cycles of treatment (Fig. 1), with a one-week interval between exposures; each treatment lasted 24 h (100% medium exchange after 24 h), as the drugs are reported to be catabolised and eliminated from the body in 24 h^78^; in particular, 77% of cumulative urinary platinum is secreted in 24 h^79^. Evaluation of chemotherapy efficacy was assessed over time by resazurin reduction capacity (at day 14 and 21 of culture, i.e., DC7 and DC14, respectively). Additionally, morphology, proliferation and apoptosis status were assessed by histology and IHC, also at DC7 and DC14. In addition, OvC-PDE from OVC26 and OVC27 samples (N = 2) were challenged with 25 or 250 µg/mL carboplatin and 10 or 100 µg/mL paclitaxel (the reported peak plasma concentrations and 10× those concentrations). Evaluation of chemotherapy efficacy was assessed at day 7 (DC0) and 21 of culture (DC14) by resazurin reduction capacity.

### Statistical analysis

One-way ANOVA statistical test or two-way ANOVA statistical test, followed by the Tukey’s multiple comparison test, were used for comparisons between more than two groups. p value were obtained by t test using Holm–Sidak method (95% confidence and statistical significance is defined using an α = 0.05). Data are shown as mean ± standard deviation of the means of N (indicated in each figure legend). Statistical analysis was carried out using GraphPad Prism 6 software for Windows (GraphPad Software, La Jolla California USA, www.graphpad.com).

### Ethics approval and informed consent

Fresh human ovarian tumours were collected from patients with signed informed consent that underwent surgery at Instituto Português de Oncologia de Lisboa, Francisco Gentil (IPOLFG). Methods were carried out in accordance with the relevant guidelines and regulations. The project was approved by the Research Council of IPOLFG and by the Ethics Committee of IPOLFG (UIC-1080 and UIC 1211). The expert drafting members are Filomena Pereira, MD (president); Cristina Nave, MD; Manuela Paiva, MD; Maria Manuela Pinto and Susana Rodrigues, MD.

## Supplementary information

Supplementary Information.

## Data Availability

The datasets used and/or analysed during the current study are available from the corresponding author on reasonable request.
