# Supplementary material for: Patient-derived ovarian cancer explants: preserved viability and histopathological features in long-term agitation-based cultures
Source: Sci Rep. 2020 Nov 10;10:19462. doi: 10.1038/s41598-020-76291-z (PMC7655823; doi:10.1038/s41598-020-76291-z)
Supplement: Supplementary file 1 — Supplementary Information. [file 41598_2020_76291_MOESM1_ESM.pdf]

# Patient-derived ovarian cancer explants: preserved viability and histopathological features in long-term agitation-based cultures

Sofia Abreu[1,2], Fernanda Silva[3], Rita Mendes[1,2], Teresa F. Mendes[1,2], Marta Teixeira[1,2], Vítor E. Santo[1,2], Erwin R. Boghaert[4], Ana Félix[3,5], Catarina Brito[1,2] *

## Supplementary Table 1: Clinical pathological annotations of ovarian cancer samples

| Case ID | Diagnosis | Age (yrs) | TMN | FIGO |
|---|---|---|---|---|
| OVC1 | High grade serous carcinoma * | 60 | ypT3bpNx (CRS3) | IIIB |
| OVC2 | Fibroma | 63 | - | - |
| OVC3 | High grade serous carcinoma | 55 | pT3CpN0 | IIIC |
| OVC4 | Endometrioid carcinoma | 59 | pT2cpN0 | IIC |
| OVC5 | Mucinous borderline tumor | 77 | pT1a | IA |
| OVC6 | Mucinous carcinoma | 79 | pT3cpNx | IIIC |
| OVC7 | Endometrioid carcinoma | 54 | pT1apN0 | IA |
| OVC8 | High grade serous carcinoma | 71 | pT3CpN1 | IIIC |
| OVC9 | High grade serous carcinoma | 69 | pT3cpNx | IIIC |
| OVC10 | High Grade serous carcinoma * | 55 | yT3c (CRS3) | IIIC |
| OVC11 | Mucinous borderline tumor | 58 | pT1a | IA |
| OVC12 | High grade serous carcinoma | 72 | pT3bpN0 | IIIB |
| OVC13 | Clear cell carcinoma | 69 | pT1c2pN0 | IC |
| OVC14 | High grade serous carcinoma | 79 | pT3cpNx | IIIC |
| OVC15 | Carcinosarcoma | 62 | pT3bpN1 | IIIB |
| OVC16 | High grade serous carcinoma | 57 | pT3cpN1b | IIIC |
| OVC17 | High grade serous carcinoma * | 68 | ypT3c (CRS –na) | IIIC |
| OVC18 | Endometrioid carcinoma | 62 | pT1a | IA |
| OVC19 | Undifferentiated carcinoma | 80 | pT3bpNx | IIIB |
| OVC20 | Low grade serous carcinoma | 39 | pT3cpN1 | IIIC |
| OVC21 | Clear cell carcinoma | 72 | pT1apN0 | IA |
| OVC23 | High grade Serous carcinoma * | 67 | pT3cpNx | IIIC |
| OVC24 | Endometrioid | 78 | pT1apN0 | IA |
| OVC25 | Mucinous borderline tumor | 60 | pT1aNx | IA |
| OVC26 | High grade serous carcinoma | 77 | pT1BNxM1 | IIIC |
| OVC27 | High grade serous carcinoma | 81 | pT3CpNx | IIIC |

* chemotherapy treatment before surgery

## Supplementary Figure 1

(A)

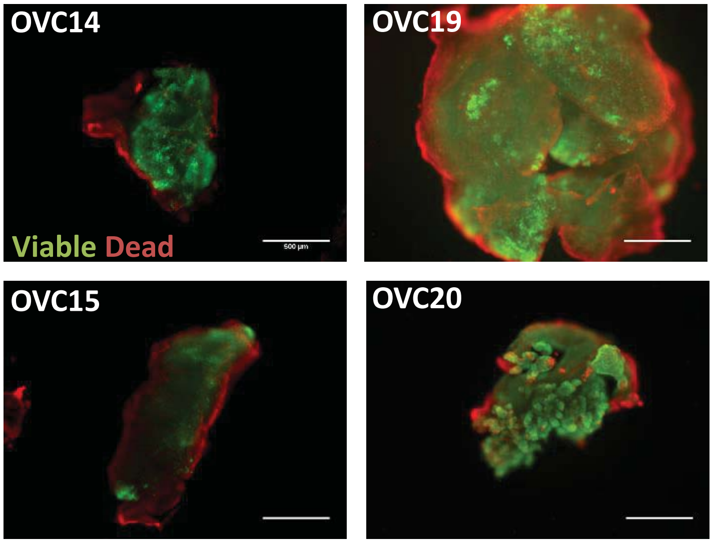


(B)

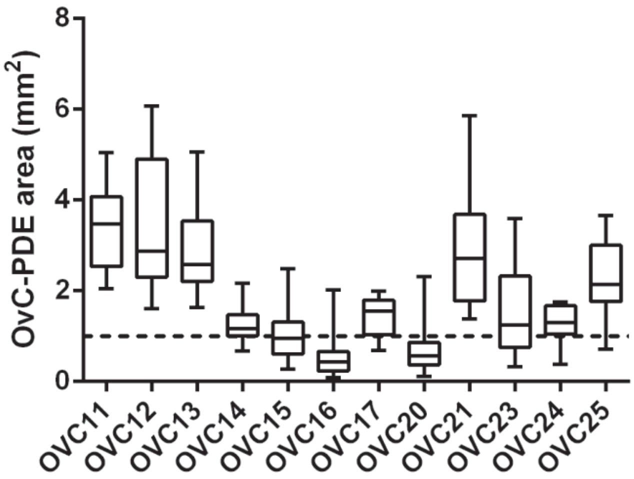

# Supplementary Figure 2

(A)

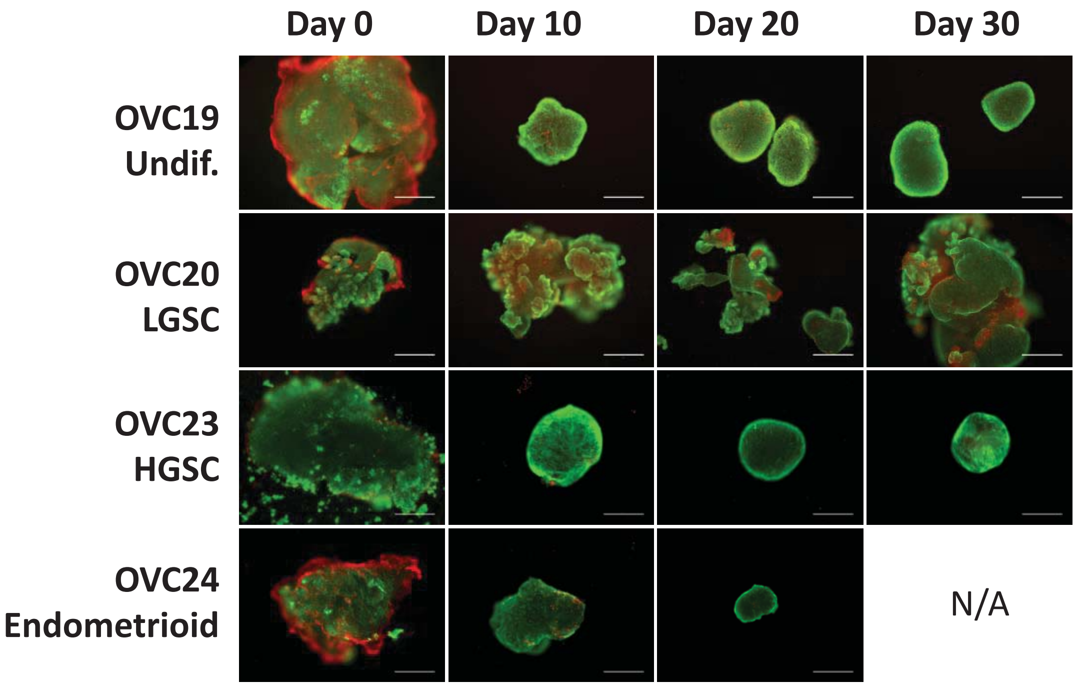


(B)

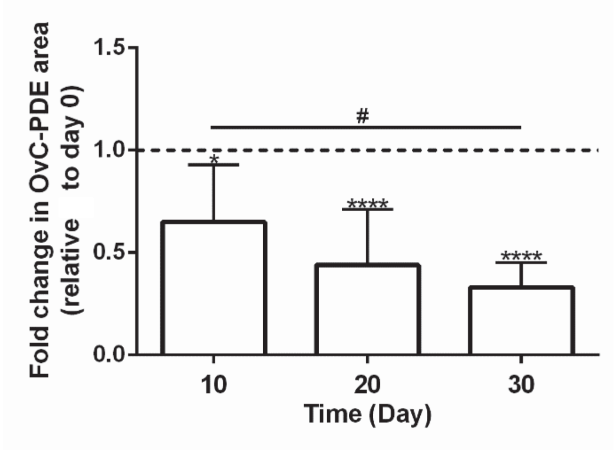


(C)

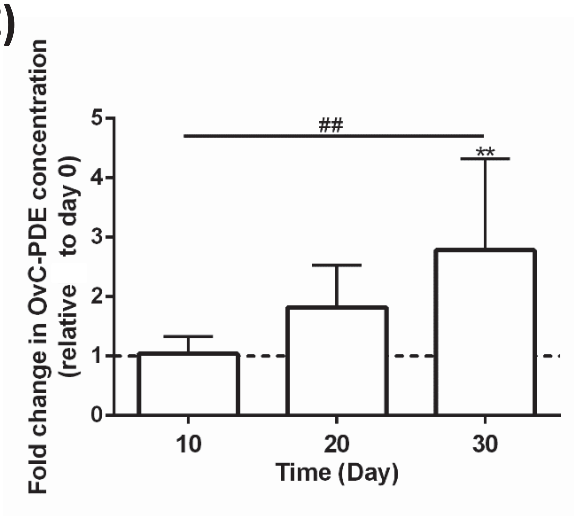

# Supplementary Figure 3

## (A)

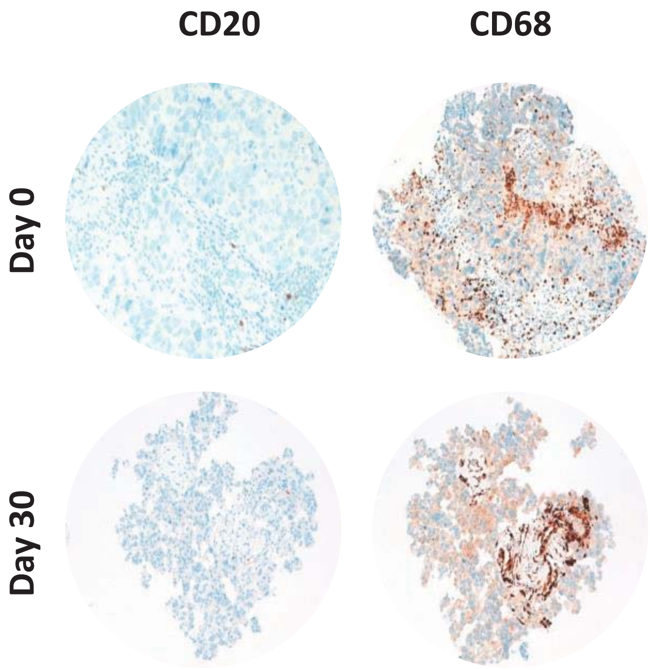


## (B)

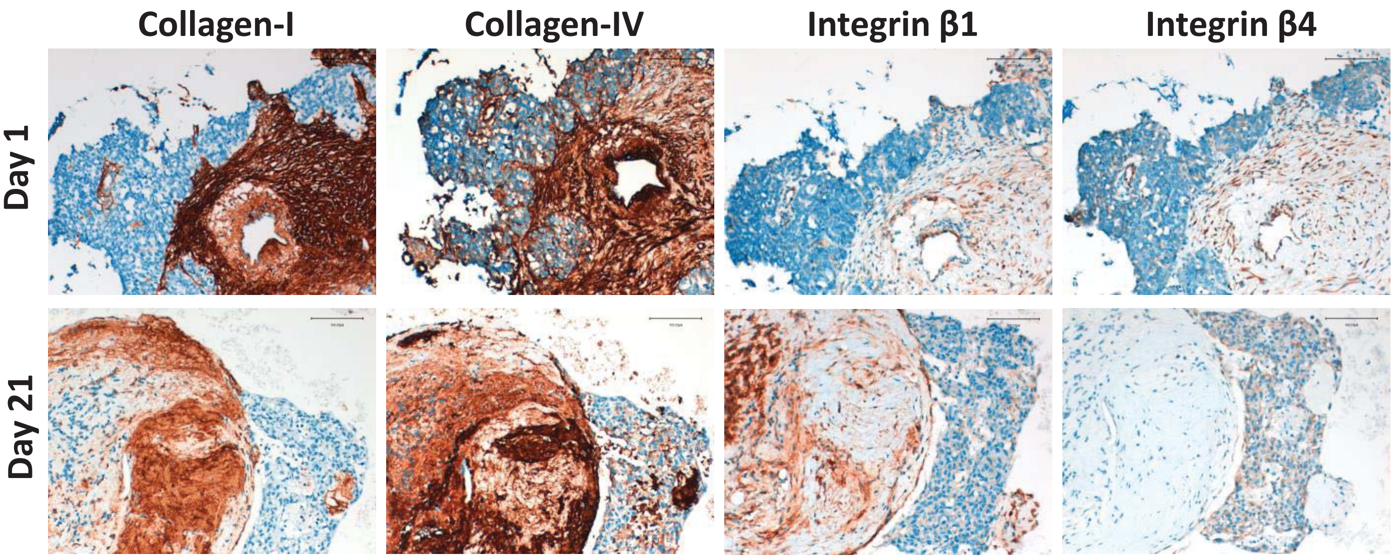

# Supplementary Figure 4

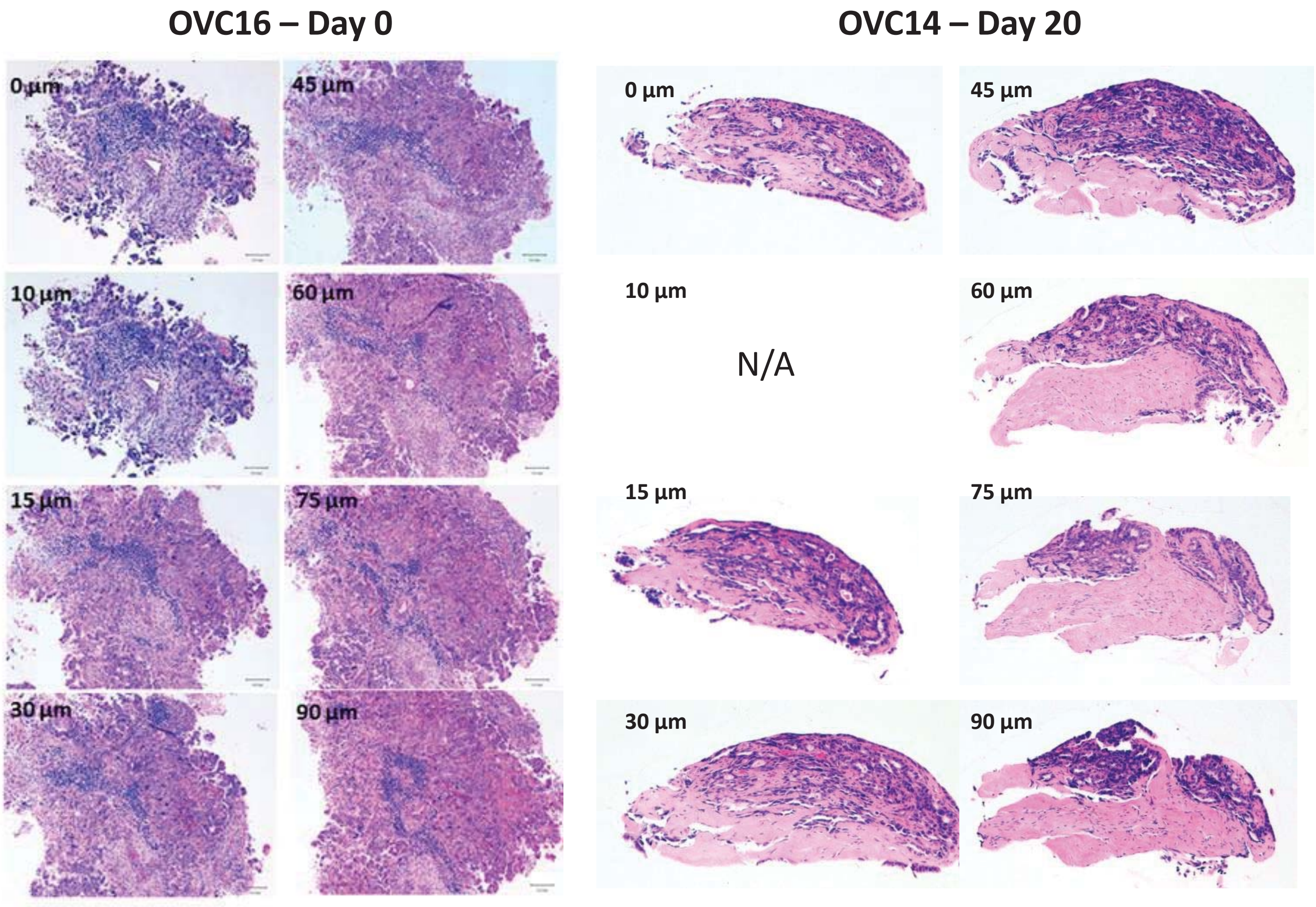

# Supplementary Figure 5

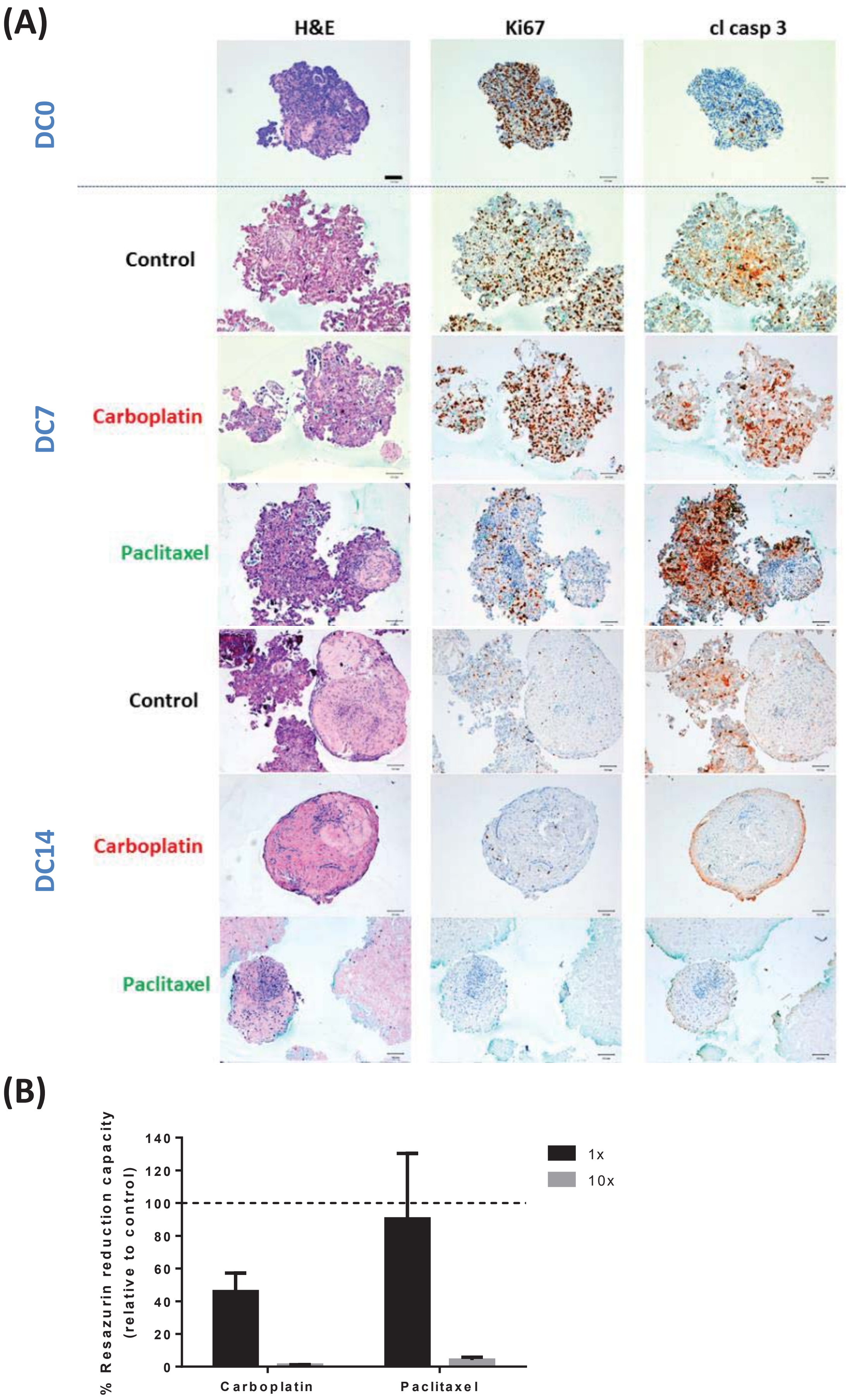

**Supplementary Figure 1: Processing of ovarian carcinoma samples for the generation of OvC-PDE cultures. (A)** Representative images (N=15) of live/dead fluorescent assay of explants, immediately after tissue processing (day 0 of culture): explants were incubated with fluorescein diacetate (FDA, green) for indication of live cells and Propidium Iodide (PI, red) for identification of dead cells. Scale bars represent 500 µm; **(B)** Explant area at Day 0 (immediately after tissue mechanical processing). Data is presented as mean ± SD (N=12, with at least 15 explants counted per tumour).

**Supplementary Figure 2: OvC-PDE cultures originated from different subtypes maintained cell viability during 30 days. (A)** Representative images (N=15) of live/dead fluorescent assay of OvC-PDE culture along time. Explants were incubated with fluorescein diacetate (FDA, green) for indication of live cells and Propidium Iodide (PI, red) for identification of dead cells. Scale bars represent 500 µm; HGSC – High Grade Serous Carcinoma, LGSC – Low Grade Serous Carcinoma; Undif., Undifferentiated carcinoma. **(B)** Explant area and **(C)** Explant concentration along OvP-PDE culture. Data is presented as mean fold-change ± SD (B, N=8; C, N=6). Two-way ANOVA statistical test (Tukey's multiple comparison test) was applied to compare the mean values at each time-point versus day 0 (*) and along culture time (#). Statistical analysis was carried out using GraphPad Prism 6 Software. # ($p<0.05$), ** or ## ($p<0.01$) and **** ($p<0.0001$).

**Supplementary Figure 3: OvC-PDE cultures maintained immune cell populations and extracellular matrix similar to the original tumor.** Immunohistochemistry analysis of cross-sections of OvC-PDE cultures from: **(A)** OVC16, at day 0 and 30. Immune markers (CD3 and CD20 for lymphocytes T and B, respectively and CD68 for macrophages). (B) OVC3, at day 1 and 21. Collagen-I, -IV and integrin β1 and β4. Scale bars represent 100 µm. Scale bars represent 100 µm

**Supplementary Figure 4: OvC-PDE cultures maintained the original cellular intra-heterogeneity.** Haematoxylin & eosin staining of cross-sections at different depths of representative OvC-PDE cultures of HGSC (OVC14 and OVC16) collected at day 0 and 20 of culture, respectively (N=2). Scale bars represent 100 µm

**Supplementary Figure 5: OvC-PDE cultures could be challenge with cycles of chemotherapy agents for evaluation drug efficacy. (A)** Haematoxylin & eosin staining and immunohistochemical analysis for proliferation (Ki67+ cells) and apoptosis (cleaved caspase 3+ cells) of representative cross-sections of OvC-PDE at day 0, 7 and 14 of drug challenge (DC) (corresponding to day 7, 14 and 21 of culture) with 25 µg/mL carboplatin or 10 µg/mL paclitaxel, N=4 (OVC15, OVC16, OVC23 and OVC24), Scale bars represent 100 µm. **(B)** Measurements of resazurin reduction capacity (in %) of the OvC-PDE along treatment time relative to OvC-PDE control culture, by performance of PrestoBlue assay. Cultures were evaluated at day 0 and 14 of drug challenge (DC) with carboplatin or paclitaxel (corresponding to day 7 and 21 of culture) (1x: 25 µg/mL carboplatin and 10 µg/mL paclitaxel; 10x: 250 µg/mL carboplatin and 100 µg/mL paclitaxel). Data is presented as a mean ± SD (N=2, OVC26 and OVC27).
